# DNA Methylation Changes in Valproic Acid-Treated HeLa Cells as Assessed by Image Analysis, Immunofluorescence and Vibrational Microspectroscopy

**DOI:** 10.1371/journal.pone.0170740

**Published:** 2017-01-23

**Authors:** Giovana M. B. Veronezi, Marina Barreto Felisbino, Maria Sílvia V. Gatti, Maria Luiza S. Mello, Benedicto de Campos Vidal

**Affiliations:** 1 Department of Structural and Functional Biology and, Institute of Biology, University of Campinas (Unicamp), Campinas, São Paulo, Brazil; 2 Department of Genetics, Evolution and Bioagents, Institute of Biology, University of Campinas (Unicamp), Campinas, São Paulo, Brazil; Peking University Health Science Centre, CHINA

## Abstract

Valproic acid (VPA), a well-known histone deacetylase inhibitor, has been reported to affect the DNA methylation status in addition to inducing histone hyperacetylation in several cell types. In HeLa cells, VPA promotes histone acetylation and chromatin remodeling. However, DNA demethylation was not checked in this cell model for standing effects longer than those provided by histone acetylation, which is a rapid and transient phenomenon. Demonstration of VPA-induced DNA demethylation in HeLa cells would contribute to understanding the effect of VPA on an aggressive tumor cell line. In the present work, DNA demethylation in VPA-treated HeLa cells was assessed by image analysis of chromatin texture, the abundance of 5-methylcytosine (5mC) immunofluorescence signals and Fourier transform-infrared (FT-IR) microspectroscopy centered on spectral regions related to the vibration of–CH_3_ groups. Image analysis indicated that increased chromatin unpacking promoted by a 4-h-treatment with 1.0 mM VPA persisted for 24 h in the absence of the drug, suggesting the occurrence of DNA demethylation that was confirmed by decreased 5mC immunofluorescence signals. FT-IR spectra of DNA samples from 1 mM or 20 mM VPA-treated cells subjected to a peak fitting analysis of the spectral window for–CH_3_ stretching vibrations showed decreased vibrations and energy of these groups as a function of the decreased abundance of 5mC induced by increased VPA concentrations. Only the 20 mM-VPA treatment caused an increase in the ratio of -CH_3_ bending vibrations evaluated at 1375 cm^-1^ in relation to in-plane vibrations of overall cytosines evaluated at 1492 cm^-1^. CH_3_ stretching vibrations showed to be more sensitive than–CH_3_ bending vibrations, as detected with FT-IR microspectroscopy, for studies aiming to associate vibrational spectroscopy and changes in DNA 5mC abundance.

## Introduction

Valproic acid (VPA), a potent anti-convulsive drug and a well-known histone deacetylase inhibitor, has been reported to induce histone hyperacetylation accompanying the decreased levels of histone deacetylases in several cell systems. Particularly in HeLa cells, an increased level of acetylation of histones H4 and H3 occurs as a function of the VPA dose or exposure period and is accompanied by chromatin remodeling [1–3].

However, the consequences of VPA treatment are not limited to changes in histone acetylation, but may also cause changes in the state of DNA methylation. A dynamic interplay between the acetylation of histone tails and changes in the abundance of DNA methylation is promoted by VPA treatment in certain cell lines such as MCF-7 human breast tumor cells, adenovirus 5 DNA-transformed HEK cells, neuroblastoma cells, lymphomonocytes, rat primary astrocytes, and lung cancer cells [4–9]. In addition, there are cell types like mouse embryonic cells and FXS lymphoblastoid cell lines in which DNA methylation levels are not affected by VPA treatment [10, 11].

When induction of chromatin unpacking was demonstrated in VPA-treated HeLa cells, effects due to DNA demethylation were not considered in addition to those concerned with histone acetylation [3]. In contrast with the relatively rapid and transient process of histone acetylation, changes in DNA methylation have a longer-standing effect [7, 12, 13]. The detection of VPA-induced DNA demethylation in HeLa cells would thus contribute to the understanding of the effect of VPA on an aggressive tumor cell line and might even inspire further studies on the mechanisms of DNA demethylation, and possible effects on promoters of tumor suppressor genes.

In the present study, our goal was to investigate whether a DNA demethylation process occurs in VPA-treated HeLa cells, as reflected by chromatin remodeling in the absence of the drug, and changes in the abundance of 5mC and in DNA infrared spectral profiles. Fourier transform-infrared (FT-IR) microspectroscopy, an analytical method that detects vibration characteristics of chemical functional groups in a sample, has been used to identify differences in DNA spectral profiles. DNA base composition and conformation, the abundance of cytosine methylation and histone binding have been associated with specific FT-IR spectral signatures [14–19]. For example, changes in the FT-IR spectral characteristics of DNA from the liver cells of non-obese diabetic mice reflect the changes in DNA methylation levels that are associated in these cells with decreased chromatin compactness and increased chromatin accessibility to MNase digestion [19]. Thus, the FT-IR spectral signature of DNA from HeLa cells should reflect changes in 5-methylcytosine (5mC) levels, if they were affected by VPA treatment. Particularly, changes should occur in the infrared spectral regions that identify the stretching and bending vibrations of–CH_3_ groups [20–24].

## Materials and Methods

### Cells

HeLa cells at passages 207/277 were incubated in a 5% CO_2_ atmosphere at 37°C and cultured in Dulbecco’s modified essential medium (DMEM, Sigma^®^, St. Louis, USA) supplemented with 10% fetal calf serum (FCS, Cultilab^®^, Campinas, Brazil) and 1% penicillin-streptomycin (Sigma^®^, 100 IU/mL and 100 μg/mL final concentrations). The cells were originally provided by the Institute Adolfo Lutz (São Paulo, Brazil) at passage 126, which had acquired them from the ATCC CCL-2 (Manassas, USA). Cells were grown in 24-well plates over round glass coverslips at a concentration of 5.0 x 10^4^ cells/mL and maintained in complete medium for 24 h. For image analysis, the cells were treated with VPA (Sigma^®^) dissolved in DMEM supplemented with 1% serum and diluted in PBS to 1 mM for 4 h and then cultivated for 24 h and 48 h in the absence of the drug. For immunofluorescence, the cells were treated with 1 mM and 20 mM VPA for 4 h. Cells cultivated in the absence of VPA were used as a control. For DNA extraction, cells were seeded for 24 h into 6-well plates at a concentration of 1.0 x 10^5^ cells/mL in complete medium, and subjected to the VPA treatments for 4 h.

### Image analysis

The cells were fixed in a mixture of absolute ethanol-glacial acetic acid (3:1, v/v) for 1 min, rinsed in 70% ethanol, air dried at room temperature, and subjected to the Feulgen reaction, specific for DNA [25], with hydrolysis conducted in 4 M HCl for 60 min at 25°C [3]. Images of the Feulgen-stained cells were obtained with a Carl Zeiss automatic scanning microspectrophotometer (Oberkochen, Germany) interfaced to a personal computer as previously described. Briefly, the operating conditions for microspectrophotometry were as follows: Planapo objective 63/0.90, optovar 2.0, measuring diaphragm diameter of 0.25 mm, field diaphragm diameter of 0.20 mm, LD-Epiplan 16/0.30 condenser, scanning spot of 0.5 μm x 0.5 μm, 100-W/12-V halogen lamp, stabilized electronic power supply, Zeiss light modulator, λ = 565 nm obtained with a Schott monochromator filter ruler, R-928 photomultiplier, and a personal computer. Individual measuring points showing ≤ 0.020 absorbance were automatically removed from the digitized nuclear image. The cutoff point of 0.100 was selected to discriminate areas covered with condensed chromatin in agreement with a previous report for HeLa cells [3]. A variable number of nuclei, dependent on the experimental conditions, was chosen at random and measured individually. The image analysis parameters used to predict the condensation profile of the chromatin were area covered with condensed chromatin relative to the nuclear area (S_C_ %) and the contrast between the average absorbance of the condensed chromatin and that of the whole chromatin (AAR, average absorption ratio) [26]. The AAR is obtained by the formula (A_C_/S_C_)/A_T_/S_T_), where A_C_ = integrated absorbance over a preselected absorbance (cutoff point); A_T_ = total integrated absorbance per nucleus; S_C_ = area in μm^2^ of the absorbing image discriminated after using the cutoff point; S_T_ = total nuclear area [26]. When the S_C_ % values are plotted against AAR values, the resulting scatter diagram reveals the position of the points that correspond to specific nuclear images [27]. Decreased S_C_ % values accompanied by increased AAR values are associated with nuclear phenotypes characterized by chromatin decondensation [3, 27–30].

### 5-methylcytosine (5mC) immunofluorescence

Cells were fixed in absolute methanol for 10 min at -20°C, washed in PBS and treated with 2 N HCl for 1 h at 37°C. The material was then washed twice in borate buffer (100 mM boric acid, 75 mM NaCl and 25 mM sodium tetraborate, pH 8.5) and blocked with 1% BSA in PBS for 1 h. Next, the cells were incubated with mouse anti-5-methylcytosine primary antibody (Sigma^®^, 1:100 diluted in 1% BSA) for 1 h at room temperature in the dark, followed by treatment with goat anti-mouse IgG conjugated to FITC (Sigma, 1:50 diluted in 1% BSA) for 1 h in the dark.

Image capture was performed using an Olympus BX60F5 microscope, QCapture and Image Pro-Plus software, and the same exposure times. ImageJ (NIH, Bethesda, USA) software was used for image analysis.

### Sample preparation for FT-IR analysis

DNA extraction was performed as previously described [31], with minor modifications. Control and VPA-treated cells were collected (for each condition, cells were pooled from three wells of a 6-well plate), centrifuged and re-suspended in cold cell lysis buffer (0.1% SDS, 10 mM Tris-HCl, pH 8.0, and 1 mM EDTA, pH 8.0). The samples were treated with proteinase K (Sigma^®^, 100 μg/mL) for 3 h at 55°C and DNase-free RNase (Thermo Scientific^®^, Waltham, USA, 33 μg/mL) for 1 h at 37°C. Proteins were precipitated by the addition of 4 M sodium acetate solution, followed by centrifugation at the maximum speed for 3 min at 4°C. The supernatant was transferred to a microcentrifuge tube containing isopropanol for DNA precipitation. The solution was centrifuged at maximum speed for 1 min at room temperature, the DNA pellet was washed three times in 70% ethanol, air dried and suspended in 0.9% NaCl solution. Approximately 12 μg of DNA was obtained for each sample, as quantified by the Qubit fluorimeter (Life Technologies^®^, Carlsbad, USA). The Thermo Scientific Nano-Drop 2000 spectrophotometer was used to assess the 260/280 absorbance ratio (~1.8), which indicates the purity of the extracted DNA. The samples were diluted to a final concentration of 364 ng/μL and were stored at -20°C until use.

Prior to the analysis of DNA preparations using FT-IR microspectroscopy, 10 μL drops of the samples dripped on slides were examined using a BX51 Olympus polarizing microscope (Olympus, Tokyo, Japan) equipped with a differential interference contrast (DIC) system and Berek’s U-CBE compensator to determine whether the extracted DNA maintained a negative birefringence and helical double-stranded conformation. This analysis was performed at the periphery of the drying drop of sample, where pure DNA crystalizes. NaCl crystals are verified only at the center of the dried drop [32]. The ambient relative humidity at which the samples were examined for optical anisotropy and FT-IR was less than 75% at 27°C. The same ambient relative humidity and room temperature were used while examining all of the DNA samples.

### FT-IR equipment/software

The FT-IR spectral acquisition of the DNA samples was performed using the Illuminat IR II^™^ microspectroscope (Smiths Detection, Danbury, CT, USA) equipped with a liquid nitrogen-cooled mercury-cadmium-telluride detector, an Olympus microscope (Olympus America) and Grams /AI 8.0 spectroscopy software (Thermo Electron Co., Waltham, MA, USA). The performance validation of the equipment was indicated by a low signal-to-noise ratio of 7929:1 [33].

The measurement site was a square of 25 μm per side. Absorbances for samples and background were obtained using 64 scans for each individual spectral profile. Absorption spectral signatures in the 3600–800 cm^-1^ wavenumber range were obtained with a resolution of 4 cm^-1^ as per the instructions from the equipment supplier. DNA samples were spread on gold-recovered slides to obtain information on DNA vibrational properties especially in the 3000–2800 cm^-1^ spectral range using an all-reflecting objective (ARO) [18, 19]. Thirteen and nine spectral profiles were obtained for the DNA from 1 mM and 20 mM VPA-treated cells, respectively, and six spectral profiles were obtained for the untreated control. Each spectral profile was subjected to baseline and level-plus-zero correction using four fitting points as provided by the OFF SET.AB application of the Grams/AI software (S1 Fig). Average spectra were then obtained for DNA from the VPA-treated and untreated control samples, followed by normalization with respect to their highest absorption peak as per the instructions provided by the Function. AB application of the Grams/AI software. Moreover, as per the Grams/AI software instructions, peak-fitting using a Gaussian function at a low sensitivity level was applied to the 2992–2850 cm^-1^ spectral window, the region assigned to ν_as_ and ν_s_ C-H stretching vibrations [24].

## Results and Discussion

### VPA induces chromatin remodeling that lasts beyond the time assigned to histone acetylation effects

**Fig 1:**
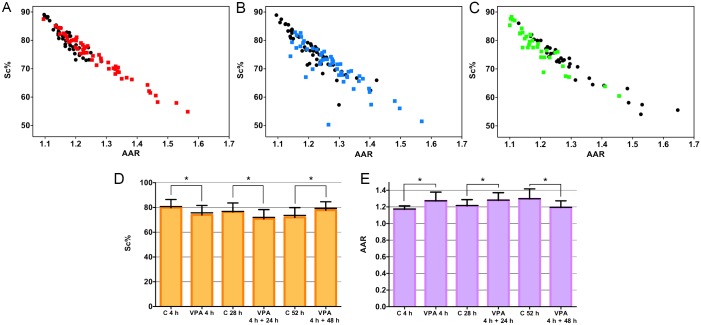
Nuclear phenotypes appear to be altered in HeLa cells in response to VPA treatment. Scatter diagrams relating of the condensed chromatin area (S_C_ %) and chromatin textural contrast (AAR) of Feulgen-stained cells. A decrease in S_C_ % values concomitant with an increase in AAR values occurred in the nuclei of cells treated with 1 mM VPA for 4 h (A, red dots). This event was maintained under conditions in which the cells were additionally cultivated in the absence of the drug for 24 h (B, blue dots) but not for 48 h (C, green dots). Black dots represent respective untreated controls. The asterisks indicate differences that are significant at P_0.05_ for comparisons of S_C_ % (D) and AAR values (E) between VPA-treated cells and respective untreated controls using the Mann-Whitney test. Error bars indicate standard deviation. The horizontal black lines of the bars represent median values. n = 50 (A, B); n = 30 (C).

Exposure of HeLa cells to 1.0 mM VPA for 4 h induced chromatin de-condensation as detected by image analysis. Scatter diagrams relating S_C_% and AAR values showed decreased S_C_% values with increasing AAR values in VPA-treated HeLa cells (Fig 1A), similar to a previous report that associated an increased level of histone acetylation with chromatin remodeling [3]. This situation persisted for 24 h (Fig 1B) after the cessation of VPA treatment but could not be sustained after a 48-h period, when chromatin packaging increased (Fig 1C). Statistical analysis confirmed the descriptive images (Fig 1D and 1E). Considering that histone acetylation is a transient mark that is rapidly reversed in the absence of a class I HDAC inducing agent like [7, 12, 13], the maintenance of textural chromatin changes in HeLa cells in the absence of the drug treatment even for 24 h suggests that another epigenetic factor such as DNA demethylation may be occurring in these cells. Even DNA demethylation, which has a longer lasting effect than histone acetylation, has been reported to be reversible in other cell systems [7], thus supporting our findings.

### VPA-induced chromatin remodeling is accompanied by a decreased abundance of DNA methylation

**Fig 2:**
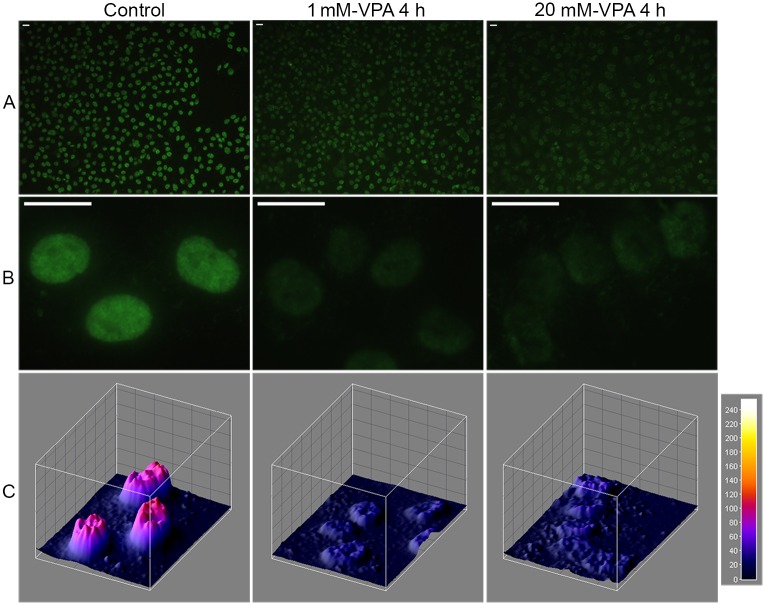
Immunofluorescence signals for DNA 5mC in VPA-treated HeLa cells. A reduction in the DNA 5mC fluorescence signals occurred with the VPA treatment (A, B). The intensity of the fluorescence signals was higher in the 20 mM-VPA-treated cells compared with the 1 mM-VPA-treated cells (A, B). Image fluorescence intensity is also shown using the ImageJ 3D plugin software (C). Arbitrary units are based on the 8-bit intensity scale (0–255). The bars equal 20 μm.

The immunofluorescence assay for 5mC demonstrated that VPA treatment drastically decreased the abundance of DNA methylation in HeLa cells (Fig 2A and 2B). In all cases, photographs were obtained using the same exposure conditions so that comparisons between samples could be made. The intensity of the signal loss was greater with the higher VPA concentration (Fig 2A and 2B), suggesting that VPA elicits a dose-dependent response [4, 7, 34]. Representative images analyzed using the ImageJ 3D plugin [35] reinforced the progressive loss of fluorescence intensity for 5mC as the VPA concentration increased compared with the untreated control (Fig 2B and 2C).

### Changes promoted by VPA in the DNA methylation status of HeLa cells affect their FT-IR spectral profiles

#### Quality attributes of the DNA samples used for the FT-IR study

**Fig 3:**
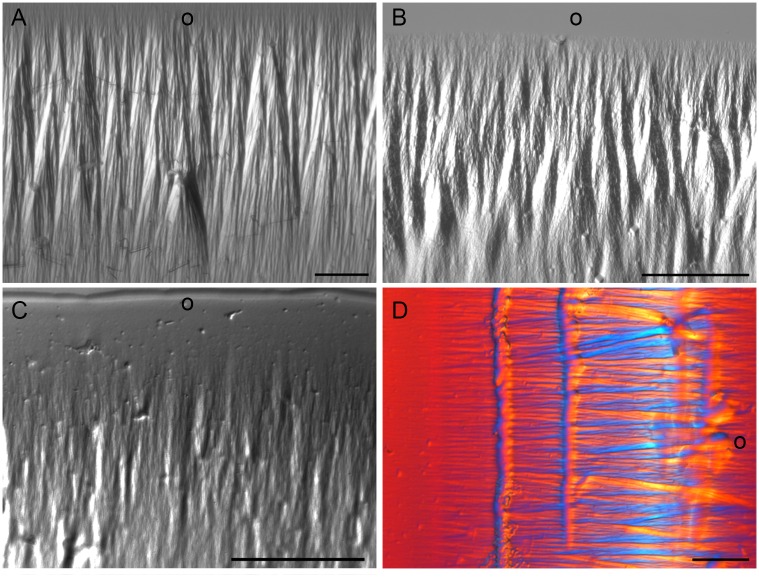
Optical anisotropy of DNA samples extracted from HeLa cells for FT-IR analysis as observed using polarization microscopy. Birefringence images of DNA from untreated control (A) and 1 mM VPA- (B) and 20 mM VPA-treated cells (C). An example of images obtained using a differential interference contrast (DIC) system is also shown for DNA from 1 mM VPA-treated cells (D). Blue and yellow interference colors against a red background (D) resulted from the orientation of the DNA fibers in relation to the gamma axis of Berek’s U-CBE compensator inserted into the microscope. This procedure was used to validate that the negative sign of the birefringence, typical of double-stranded B-DNA, was achieved. The outer edge of the DNA drops dried on slides is indicated (o). The bars equal 50 μm.

**Fig 4:**
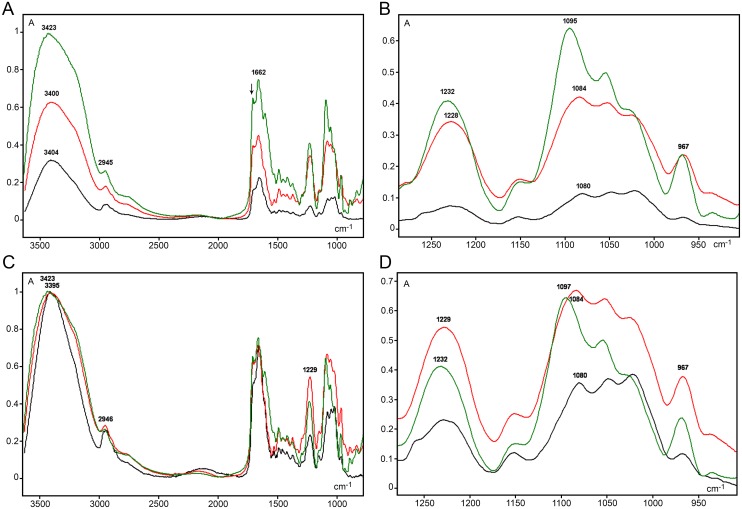
FT-IR average spectral profiles for DNA from 1 mM VPA-treated (red line), 20 mM VPA-treated (olive line), and untreated (black line) HeLa cells. Non-normalized spectra (A, B) and spectra normalized with respect to the highest band peak (C, D) are shown. (B) and (D) are spectral details from (A) and (C), respectively. The arrow in (A) indicates ~1707 cm^-1^ wavenumber position. Small A, absorbances.

The integrity of the double-stranded DNA studied here using FT-IR analysis was demonstrated by optical anisotropic data (negative birefringence) (Fig 3) and by the presence of an FT-IR spectral band peak at 1232–1225 cm^-1^ (Fig 4A–4D) and a shoulder at 1707 cm^-1^ (Fig 4A and 4C). The band peak at 1232–1225 cm^-1^ is not only associated with the DNA PO_2_^-^ antisymmetric stretching vibration (ν_as_) but it is also sensitive to the DNA molecular geometry [Taillandier *et al*. 1985 –*apud* 22] and a mark of the DNA *B*-form [36, 37]. The absorption shoulder at 1707 cm^-1^ suggests that the DNA extracted from VPA-treated and untreated cells was not denatured [22, 38]. In addition, band peaks at 1080 cm^-1^, 1084 cm^-1^ and 1097–1095 cm^-1^ for the untreated control, 1 mM and 20 mM VPA-treated cell samples, respectively, is probably related to PO_2_^-^ symmetric vibration (ν_s_), although the absorption peak for the DNA from the 20 mM VPA-treated cells was shifted to a lower frequency (Fig 4B and 4D). This band peak was also always lower than the peak corresponding to ν_as_ PO_2_^-^ (Fig 4B and 4D), which is in agreement with a ν_as_ PO_2_^-^/ ν_s_ PO_2_^-^ ratio < 1.0 that is expected for pure DNA samples [17, 22].

#### VPA-induced changes in the DNA methylation status of HeLa cells affect their FT-IR band peaks associated with–CH_3_ stretching vibrations

**Fig 5:**
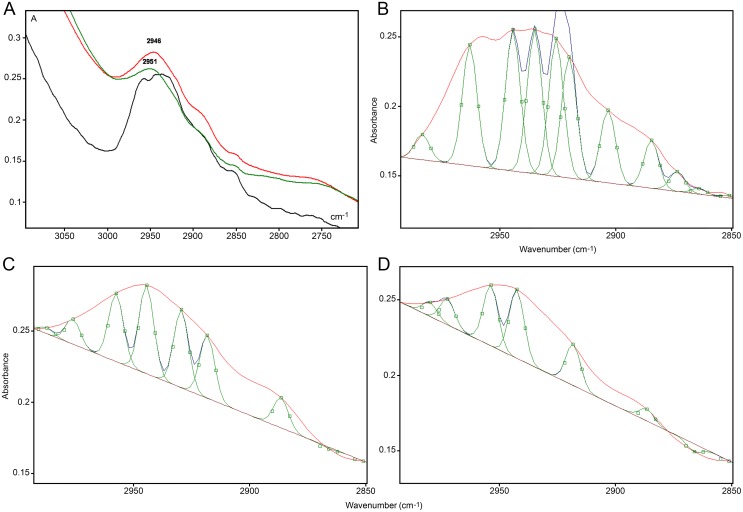
Details of the IR spectral window in the 2990–2850 cm^-1^ wavenumber range. The band peak in (A) is shown as originally obtained from normalized spectra for DNA from 1 mM VPA-treated (red line), 20 mM VPA-treated (olive line) and untreated (black line) cells. The same FT-IR spectral profiles after peak fitting using Grams/AI 8.0 software, Gaussian function and low sensitivity level are shown for the untreated sample (B), the 1 mM VPA-treated cells (C), and the 20 mM VPA-treated cells (D). Original traces appear as red lines, and fitted peaks are represented as green lines (B-D). Small A, absorbances.

In the ~2992–2850 cm^-1^ spectral region, the peak assigned to the ν_as_ and ν_s_ C-H stretching vibrations in 5mC methyl groups [19] was evident in all cases (Figs 4A and 4C and 5A–5D) but was less elevated in the DNA spectrum from VPA-treated cells (Fig 5A). When a peak fitting procedure was applied to this spectral window, the band peak was resolved into different peaks (Fig 5B–5D; Table 1). The most prominent peak for the DNA from VPA-treated cells appeared slightly shifted to longer frequencies compared with the untreated control, although practically no difference occurred as a function of the VPA treatment dose used (Table 1). However, it is evident that the total area of the band peak, as well as the area of the main fitted peak, decreased with increasing VPA concentrations (Table 1). The total band area decrease was verified even when non-normalized spectral curves were compared (control, 1.5235 units; 1 mM VPA-treated cells, 1.2453 units; 20 mM VPA-treated cells, 1.1572 units). In IR spectroscopy, a decrease in area under an absorption band peak has been related to a decrease in absorbed energy [18, 39, 40]. In the present case, this decrease was probably associated with the lower abundance of cytosine methylation induced by VPA treatment (Fig 2) similar to data from hyperglycemic non-obese diabetic mice [19]. Differences in the number of peaks provided by the peak fitting process suggest that changes in the chemical environment at the level of stretching vibrations of–CH_3_ groups occurred with changes in DNA methylation levels [19].

**Table 1:** Statistics for the FT-IR band peak related to–CH_3_ stretching vibrations.

Cell treatments	Number of peaks	Main peak frequency (cm^-1^)	Main peak area units	Total area units
Untreated control	11	2935	0.8255	4.8044
1 mM VPA	8	2944	0.4880	1.9798
20 mM VPA	6	2942	0.3623	1.1720

Peak fitting provided by Grams/AI 8.0 software (function: Gaussian; sensitivity: low).

Wavenumber edges: 2992 and 2850 cm^-1^

As expected from the analysis of DNA FT-IR spectral profiles using the ARO objective [18, 19], the most elevated band peaks in the 3600–800 cm^-1^ spectral range were verified at the 3425–3395 cm^-1^ wavenumber region for VPA-treated and untreated cells (Fig 4A and 4C). This region has been assigned to -NH and–NH_2_ group stretching vibrations and hydrogen bonding [21, 23, 24, 41].

Although the PO_2_^-^ symmetric vibration at the 1080 cm^-1^ wavenumber is important to differentiate stem cells in human intestinal crypts of tissue preparations, assuming an association with DNA conformational changes and chromatin remodeling [42], extrapolation of this report to interpretation of the present spectra obtained for isolated DNA preparations from HeLa cells could not be currently established.

#### IR spectral regions corresponding to overall cytosine in-plane vibrations and–CH_3_ bending vibrations are less sensitive at reflecting DNA methylation changes in HeLa cells

**Fig 6:**
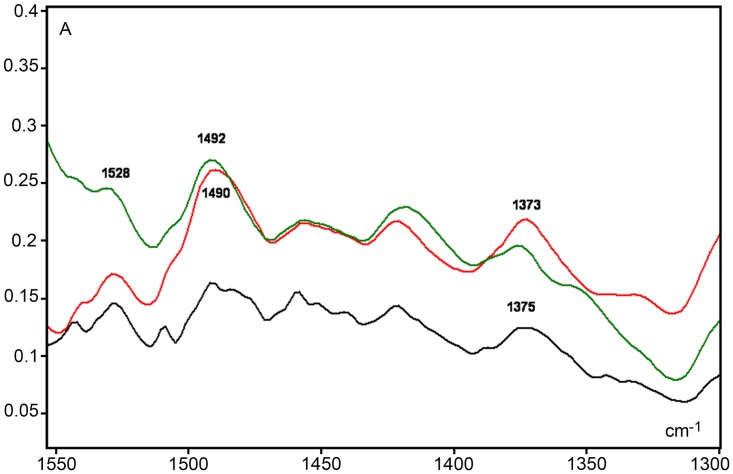
Details of the IR spectral window in the 1530–1350 cm^-1^ range. Spectral profiles are shown for DNA from 1 mM VPA-treated (red line), 20 mM VPA-treated (olive line) treated and untreated (black line) cells. A, absorbances.

In the fingerprint spectral region, peak absorbances were observed at 1492 cm^-1^ and 1375 cm^-1^ frequencies (Fig 6), which may be due to the contribution of overall cytosine in-plane vibrations [22, 37, 43, 44] and -CH_3_ bending vibrations [24], respectively. However, the intensity of these peaks appears increased in the DNA spectra from VPA-treated cells even after normalization of the spectral curves (Fig 6). On the other hand, if a ratio is calculated for absorbances at 1375 cm^-1^ in relation to the absorbances obtained at 1492 cm^-1^, its value is higher for 20 mM VPA-treated cells (1.3799) than for the 1 mM VPA-treated cells (1.2034) and the untreated control cells (1.3224). Moreover, these data were compatible with the decreased abundance of 5-methylcytosine revealed in the immunofluorescent images (Fig 2). However, this rationale did not apply to the comparison of ratios between the 1 mM VPA-treated cells and the untreated cells. Considering that at this spectral region absorptions of different chemical groups and bending vibrational motion types may overlap [24], it is not recommended for the association of DNA 5mC abundance and VPA demethylation effects at least in HeLa cells.

A band peak at 1662–1661 cm^-1^, which has been attributed to the contribution of thymine and adenine bases [18, 22, 37], was evident and similar among all of the spectra we compared (Fig 4A and 4C)

## Conclusions

1. Changes in the DNA methylation status of HeLa cells induced by VPA were suggested by the results of image analysis of chromatin texture and confirmed by decreased 5mC immunofluorescence signals. These changes also affected the FT-IR spectral profiles of the DNA. In particular, the intensities and frequencies corresponding to ν_as_ and ν_s_ -CH_3_ stretching vibrations decreased as the concentration of VPA increased, in agreement with a decrease in abundance of 5mC. The FT-IR results are supported by previously reported changes in FT-IR spectra for non-obese diabetic mice displaying decreased levels of DNA methylation under hyperglycemic conditions [19].
2. Based on the calculation of a ratio of absorbances at 1492 cm^-1^/1375 cm^-1^ (-CH_3_ group bending vibrations in relation to the contribution of overall cytosine in-plane vibrations), the increased value for the 20 mM VPA-treated sample-DNA agreed with the decreased abundance of methylated cytosine. However, this association did not apply to the DNA obtained from cells treated with 1 mM VPA, possibly because of the interference of the vibrational effects of other functional groups at this spectral region.
3. For further studies that aim to associate vibrational spectroscopy and changes in DNA 5mC abundance, analysis of the IR spectral region concerned with the frequency of -CH_3_ stretching vibrations is more indicated than that assigned to–CH_3_ bending and cytosine in-plane vibrations.

## Supporting Information

S1 Fig Baseline-corrected raw FT-IR spectral profiles for DNA from untreated (A), 1 mM VPA-treated (B) and 20 mM VPA-treated (C) HeLa cells. X axis, absorbances (A); Y axis, wavenumbers in cm^-1^.
(TIF)
