# Supplementary figures and images for: DNA Methylation Changes in Valproic Acid-Treated HeLa Cells as Assessed by Image Analysis, Immunofluorescence and Vibrational Microspectroscopy
Source: PLoS One. 2017 Jan 23;12(1):e0170740. doi: 10.1371/journal.pone.0170740 (PMC5256918; doi:10.1371/journal.pone.0170740)

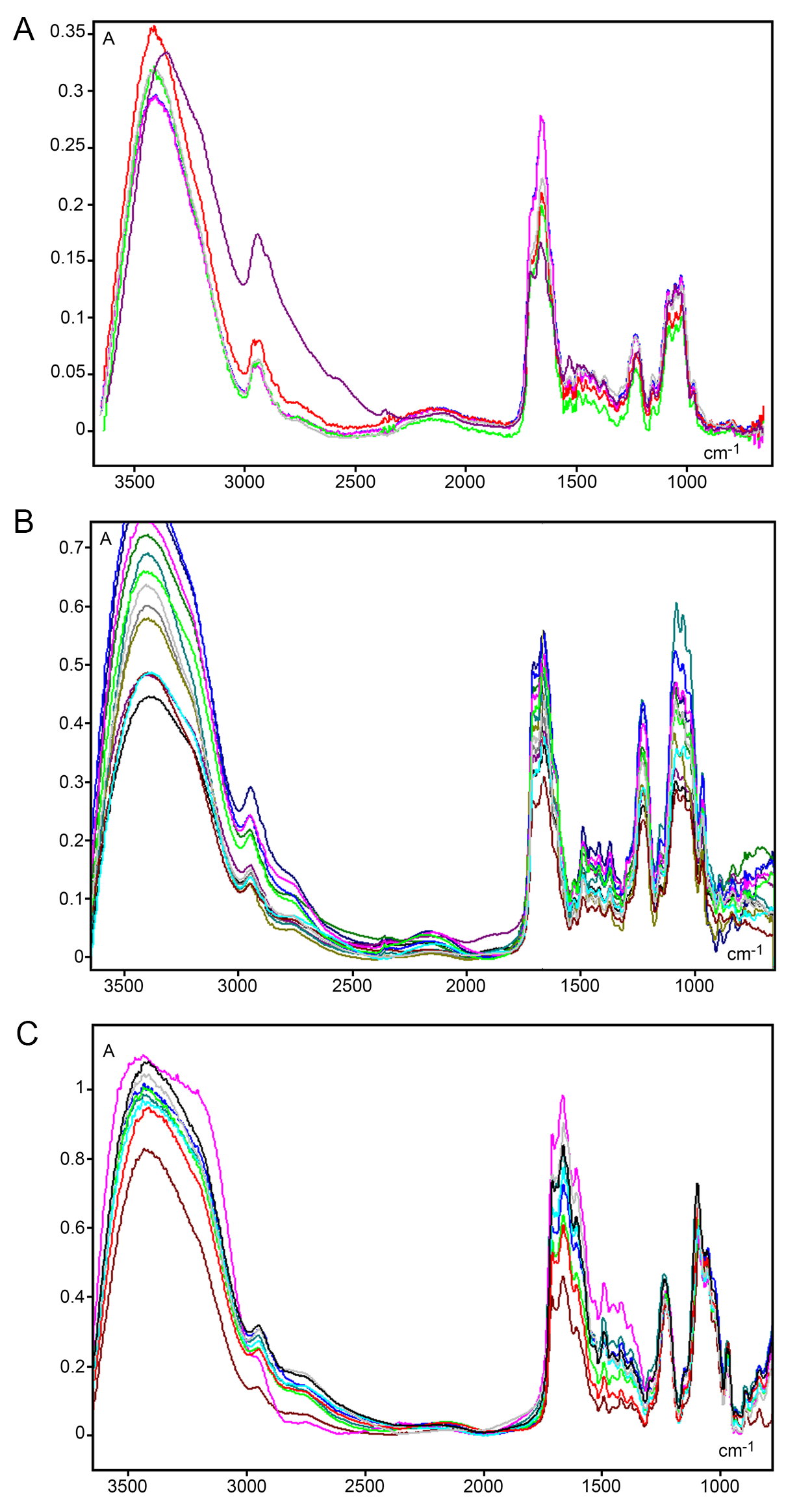

Supplement: S1 Fig — X axis, absorbances (A); Y axis, wavenumbers in cm-1. (TIF) [file pone.0170740.s001.tif]
